# Supplementary material for: FLIP(C1orf112)-FIGNL1 complex regulates RAD51 chromatin association to promote viability after replication stress
Source: Nat Commun. 2024 Jan 29;15:866. doi: 10.1038/s41467-024-45139-9 (PMC10825145; doi:10.1038/s41467-024-45139-9)
Supplement: Supplementary file 1 — Supplementary Information [file 41467_2024_45139_MOESM1_ESM.pdf]

SUPPLEMENTARY FIGURES

FLIP(C1orf112)-FIGNL1 Complex Regulates RAD51 Chromatin Association to Promote Viability After Replication Stress

Jessica D. Tischler[1,#], Hiroshi Tsuchida[1,#], Rosevalentine Bosire, Tommy T. Oda[1,2], Ana Park[1,2], and Richard O. Adeyemi[1,3]*

[1]Basic Sciences Division, Fred Hutchinson Cancer Center, Seattle, WA 98109
[2]University of Washington, Seattle WA 98195
[3]Molecular and Cellular Biology program, University of Washington, Seattle WA 98195

# These authors contributed equally.

* Correspondence: radeyemi@fredhutch.org

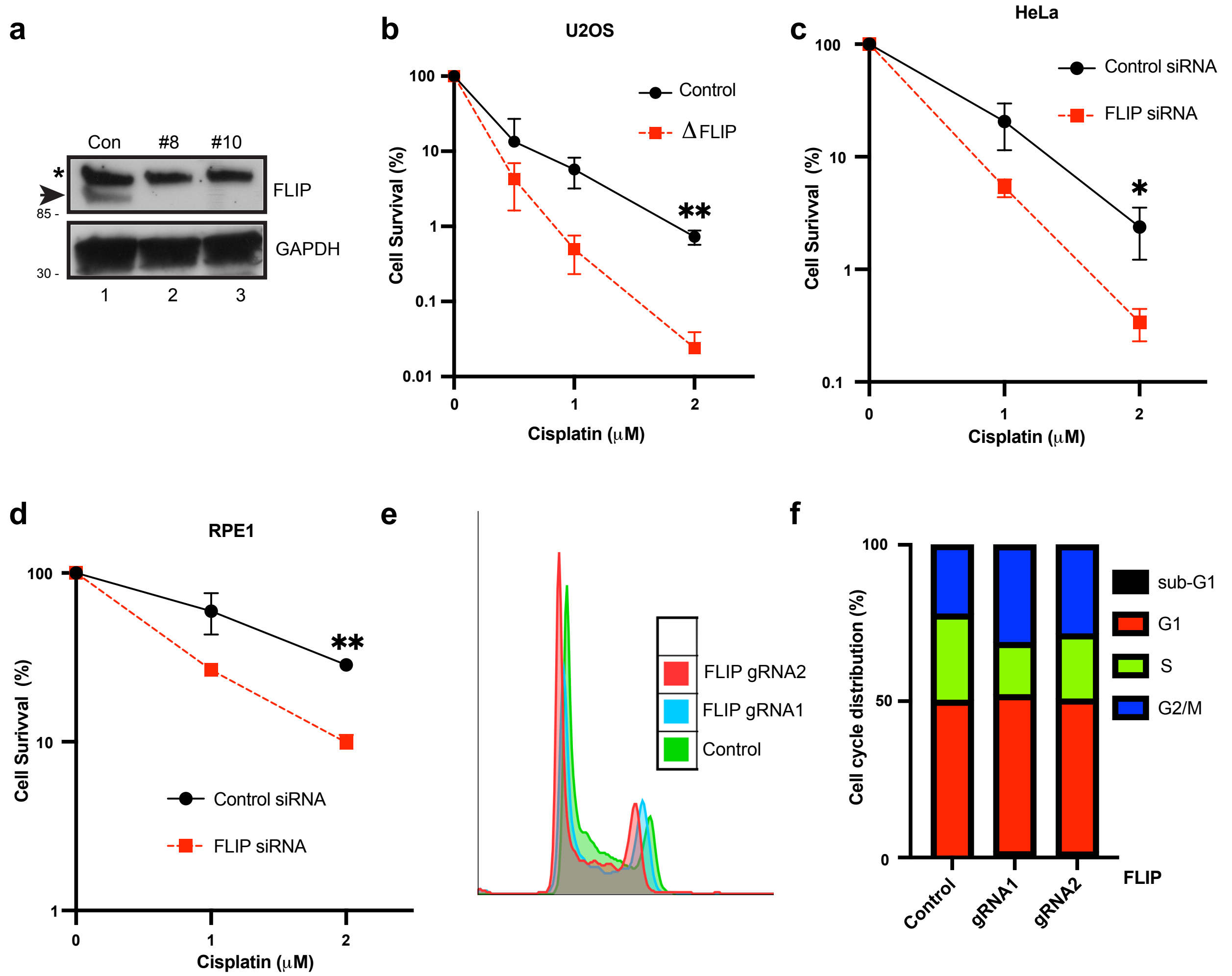

Supplementary Figure 1.

(a) Western blots showing loss of FLIP in both FLIP knockout clones (#8 and #10). Con – control gRNA expressing U2OS cells. **(b)** Colony survival assays (CSAs) showing survival of U2OS cells expressing control gRNA or FLIP gRNA knockout upon treatment with indicated doses of cisplatin. Mean ± SD shown, n=3 independent experiments **(c)** CSAs showing survival of control or FLIP siRNA - treated HeLa cells upon treatment with indicated doses of cisplatin. Mean ± SD shown, n=3 independent experiments. **(d)** CSAs showing survival of control or FLIP siRNA - treated RPE1 cells upon treatment with indicated doses of cisplatin. Mean ± SD shown, n=2 independent experiments. For **b-d**, *$P \leq 0.05$; **$P \leq 0.01$; t-tests (two-tailed) of the indicated dose. **(e-f)** Cell cycle distribution in control or FLIP gRNA expressing U2OS cells overlayed in the absence of drug treatment, quantified in (F). N=3, representative experiment shown. Source data are provided as a Source Data file.

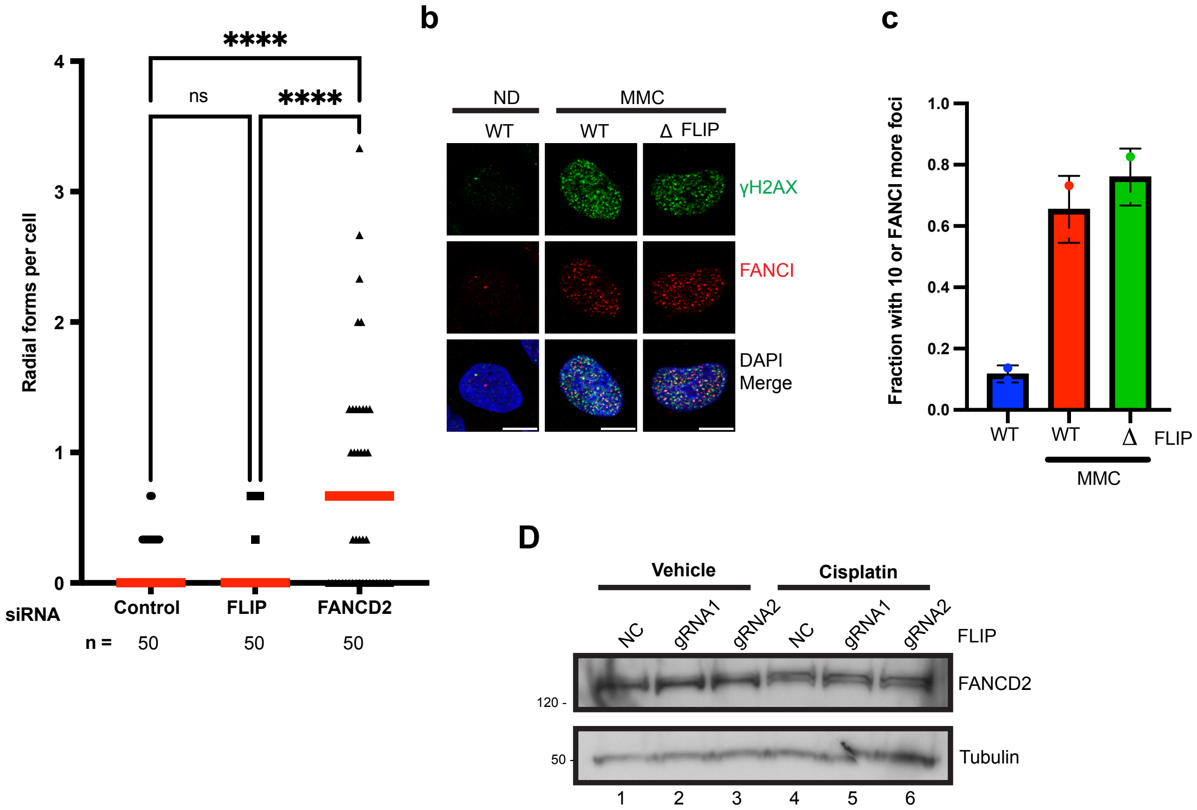

Supplementary Figure 2.

(a) Numbers of radial form chromosomes at metaphase in MMC-treated HeLa cells transfected with indicated siRNAs. Red bar is median of each group, n = 50, three independent experiments. ****$P \leq 0.0001$**,** ns – not significant. Kruskal-Wallis test followed by Dunn's. **(b)** U2OS cells expressing control or FLIP gRNA were treated with 1 μM MMC for 8 hours prior to IF analyses for FANCI and γH2AX foci. **(c)** Quantification of the experiment in (B). Mean ± SD shown, n = 2 independent experiments. **(d)** Western blot analyses showing unchanged FANCD2 ubiquitination following cisplatin treatment in U2OS cells expressing two different gRNAs to FLIP or control gRNA (NC). Scale bars = 10 μm. Source data are provided as a Source Data file.

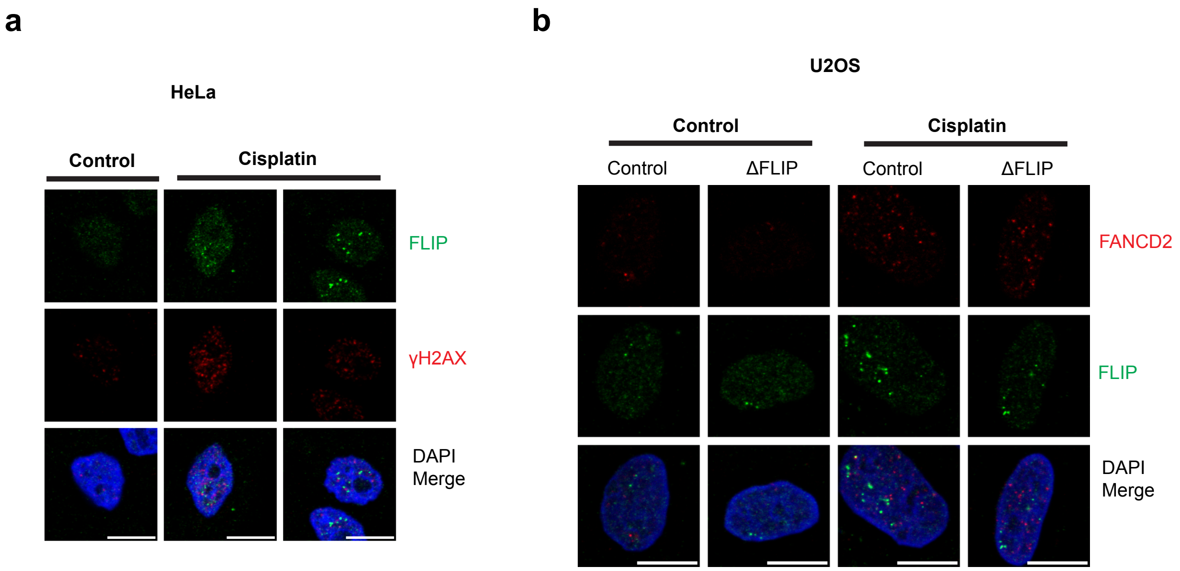

Supplementary Figure 3.

(a) IF images showing recruitment of GFP-FLIP to foci in HeLa cells after treatment with 0.5 μM cisplatin for 16 h. Cells were pre-extracted to remove soluble protein. **(b)** IF images in U2OS cells showing recruitment of GFP-FLIP to foci that only partly colocalize with FANCD2 after treatment with 0.5 μM cisplatin for 16 h. Cells were pre-extracted to remove soluble protein. Scale bars = 10 μm.

a

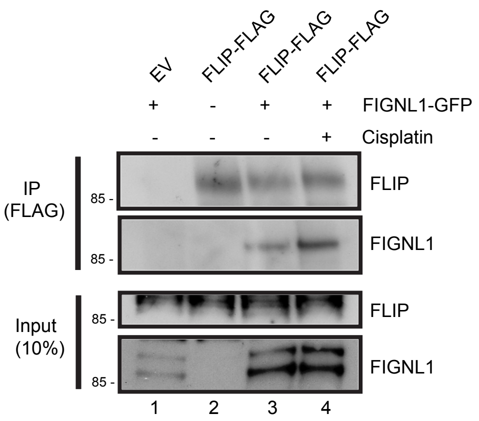

Supplementary Figure 4.

(a) 293T cells were transfected with EV, FLAG-FLIP or GFP-FIGNL1 as indicated for 48 h. Cells were then treated with 2 μM cisplatin for 24 h prior to coIP after lysate treatment with benzonase. Source data are provided as a Source Data file.

a

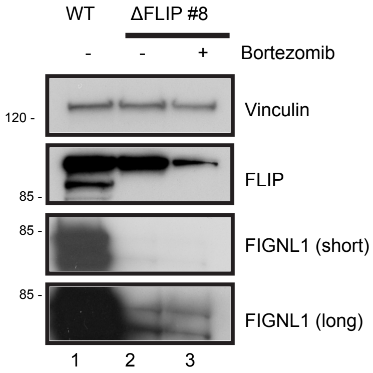

Supplementary Figure 5.

(a) Control gRNA expressing U2OS cells (WT) or ∆FLIP knockouts were treated with vehicle or 10 Bortezomib for 14 hrs as indicated. WCLs were prepared and assayed for the indicated proteins by WB. Source data are provided as a Source Data file.

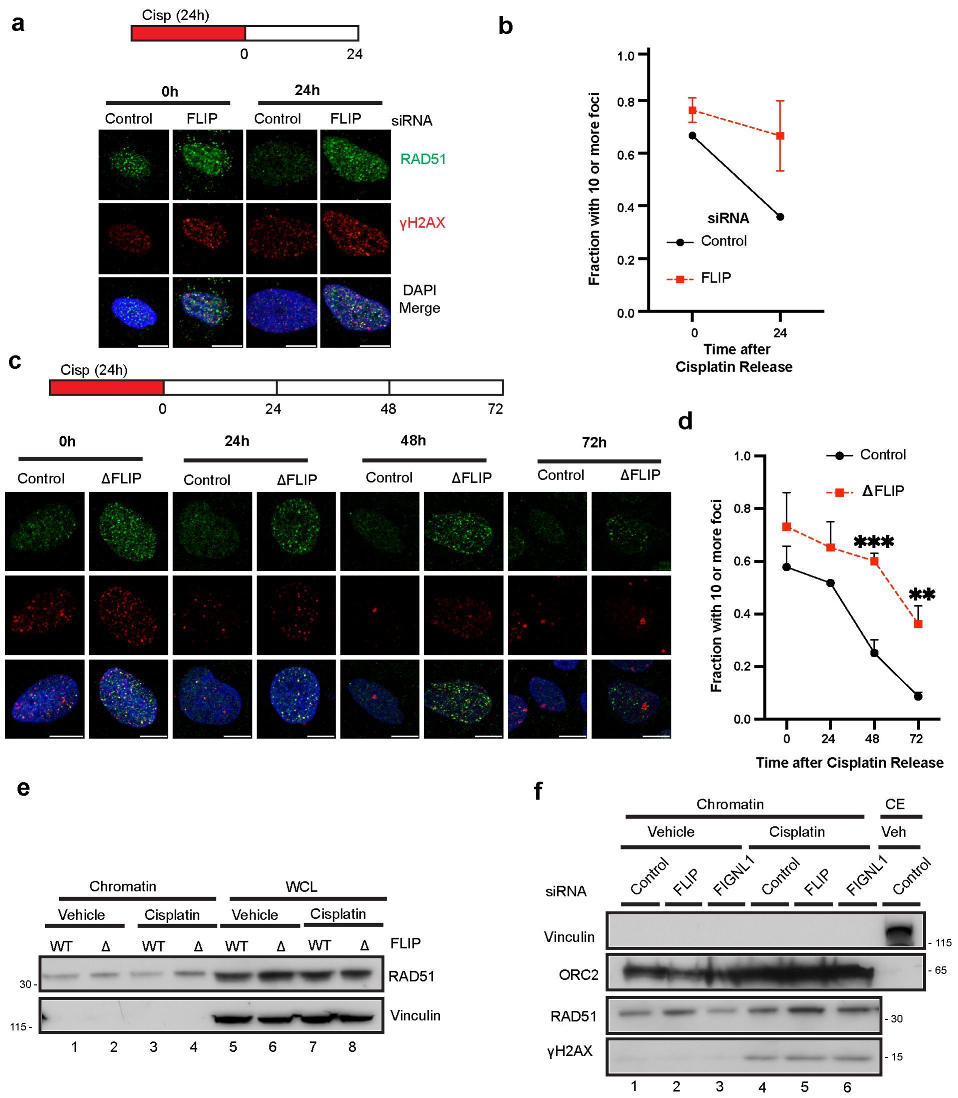

Supplementary Figure 6.

(a) Top: Schematic of the experiment. U2OS cells were transfected with the indicated siRNAs for 48 h prior to 1 μM cisplatin treatment as indicated. Bottom: Representative IF images show RAD51 and γH2AX foci at different time points after release from cisplatin treatment. **(b)** Quantification of the experiment in (A), mean ± SD shown, n=2 independent experiments. **(c)** Top: Schematic of the experiment. Control gRNA expressing or ΔFLIP U2OS cells were treated with 0.5 μM cisplatin as indicated. Bottom: Representative IF images show RAD51 and γH2AX foci at different time points after release from cisplatin treatment. **(d)** Quantification of the experiment in (c), mean ± SD shown, n=3 independent experiments. Statistics represent unpaired t-tests (two-tailed) for each time point, $**P = 0.0025$; $***P = 0.0005$. **(e)** Control gRNA-expressing (WT) or ΔFLIP U2OS cells were treated with vehicle or 1.5 μM cisplatin for 18 h. Samples were split 2:1 ratio then processed for chromatin fraction or WCLs prior to blotting for the indicated proteins. **(f)** U2OS cells were reverse transfected with control, FLIP or FIGNL1 siRNAs for 48h prior to treatment with vehicle or 2.5 μM cisplatin for 18 h. Cells were fractionated and blotted for the indicated proteins. Scale bars = 10 μm. Source data are provided as a Source Data file.

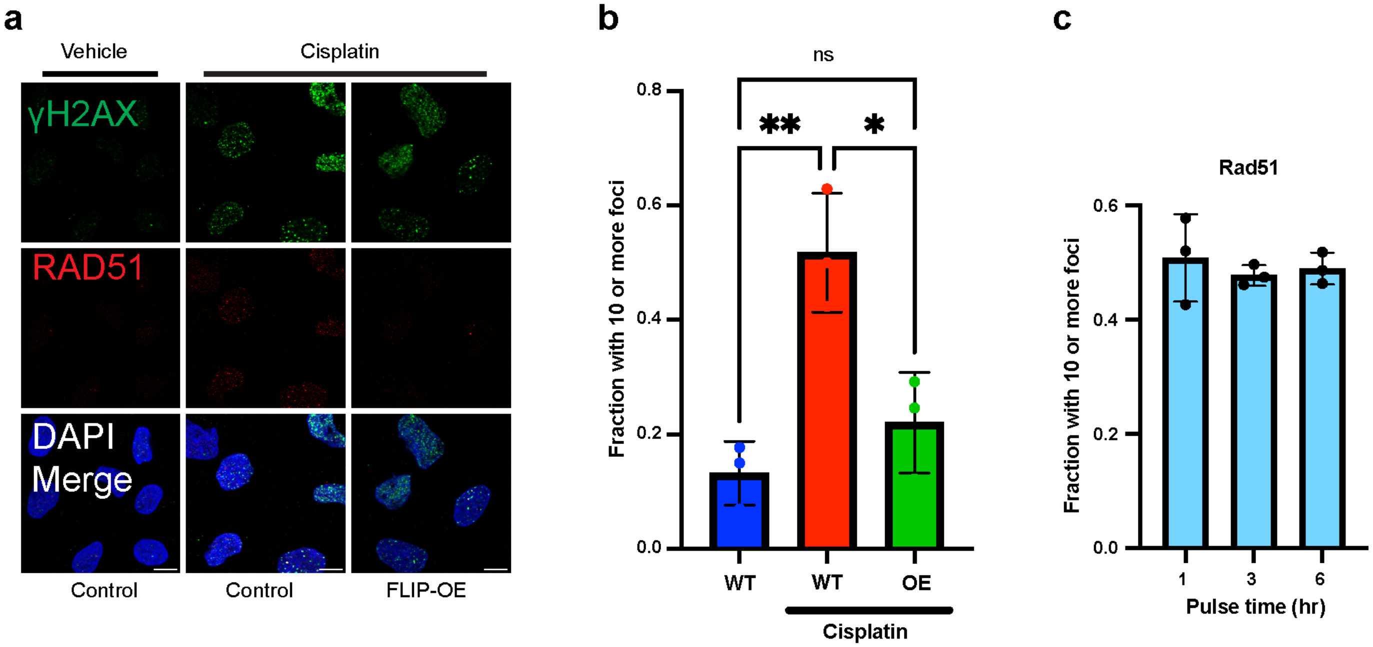

Supplementary Figure 7.
(a) Control or FLIP-GFP overexpressing U2OS cells were treated or not with 0.5 μM cisplatin for 18 hr as indicated. Representative IF images show RAD51 and γH2AX foci. **(b)** Quantification of (A). Mean ± SD shown, n = 3 independent experiments, ordinary one-way ANOVA followed by Tukey's, ns – not significant ($P = 0.4605$); *$P = 0.0122$; **$P = 0.0035$. **(c)** Quantification of percent EdU positive cells, mean ± SD shown, n=3 independent experiments. Scale bars = 10 μm. Source data are provided as a Source Data file.

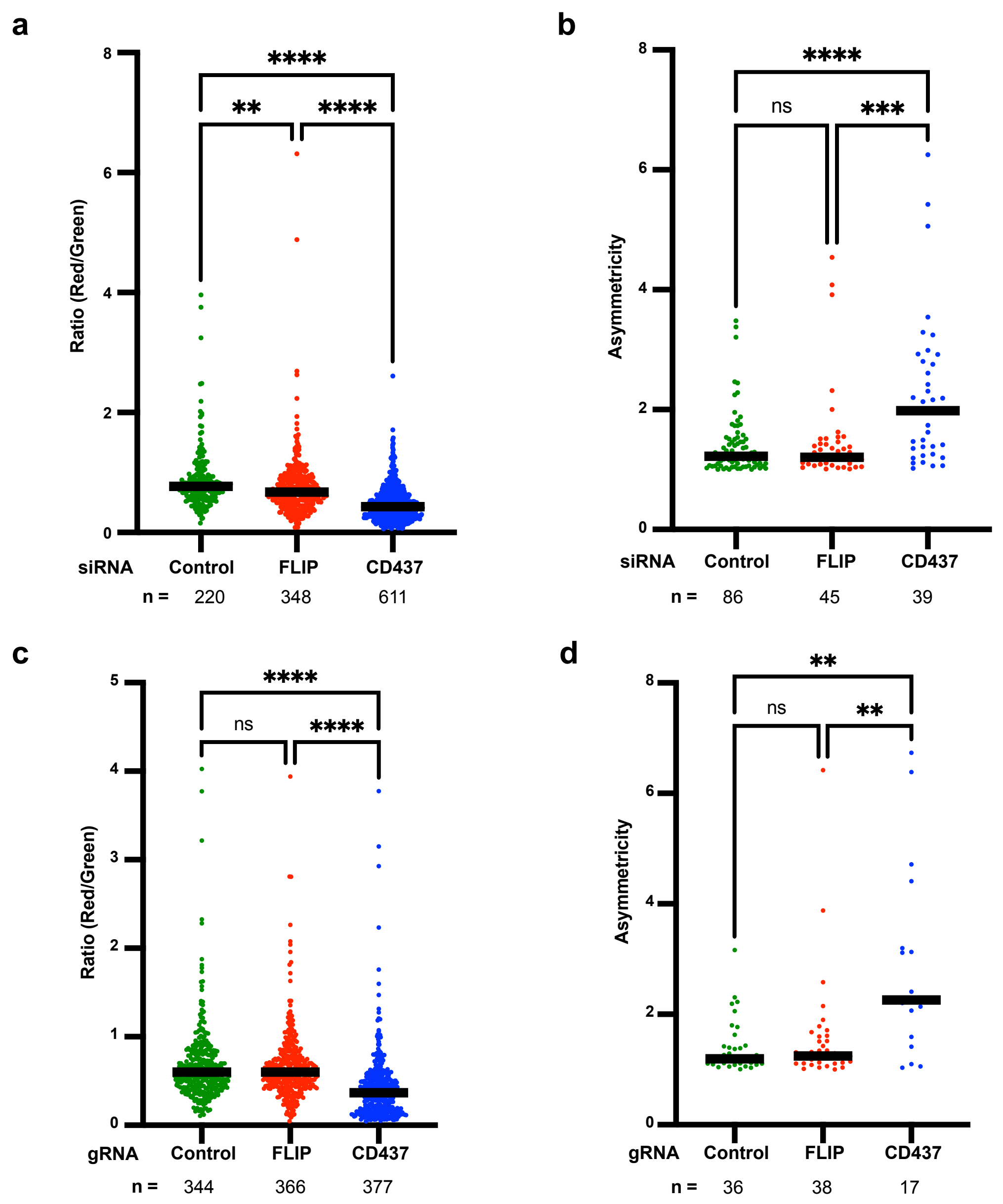

Supplementary Figure 8.

(a) Ratios (red/green) of the length of two-color DNA fibers obtained from U2OS cells transfected with siRNAs as indicated or treated with CD437. **(b)** Quantified asymmetricity of the bidirectionally labeled DNA replication fork from DNA fiber assay. The ratios of the length of two red fibers in three-segmented DNA fibers (red-green-red) were calculated (long red/short red). DNA fibers were obtained from U2OS cells transfected with siRNAs as indicated or treated with CD437. **(c)** Same as (a). DNA fibers were obtained from U2OS cells expressing indicated gRNAs, or control gRNA with CD437 treatment. **(d)** Same as (b). DNA fibers were obtained from U2OS cells expressing indicated gRNAs, or control gRNA with CD437 treatment. **(a-d)** Black line represents median, three independent experiments, representative experiment shown.
