# Supplementary material for: FLIP(C1orf112)-FIGNL1 complex regulates RAD51 chromatin association to promote viability after replication stress
Source: Nat Commun. 2024 Jan 29;15:866. doi: 10.1038/s41467-024-45139-9 (PMC10825145; doi:10.1038/s41467-024-45139-9)
Supplement: Supplementary file 3 — Description of Additional Supplementary Files [file 41467_2024_45139_MOESM3_ESM.pdf]

Description of Additional Supplementary Files

File Name: Supplementary Data 1
Description: Overlap of top U2OS and RPE genome-wide cisplatin screen hits.

File Name: Supplementary Data 2
Description: List of primers and gene fragments used.
