# Supplementary material for: FLIP(C1orf112)-FIGNL1 complex regulates RAD51 chromatin association to promote viability after replication stress
Source: Nat Commun. 2024 Jan 29;15:866. doi: 10.1038/s41467-024-45139-9 (PMC10825145; doi:10.1038/s41467-024-45139-9)
Supplement: Supplementary file 6 — Reporting Summary [file 41467_2024_45139_MOESM6_ESM.pdf]

nature portfolio

Corresponding author(s): Richard Adeyemi

Last updated by author(s): Jan 5, 2024

Reporting Summary

Nature Portfolio wishes to improve the reproducibility of the work that we publish. This form provides structure for consistency and transparency in reporting. For further information on Nature Portfolio policies, see our Editorial Policies and the Editorial Policy Checklist.

Statistics

For all statistical analyses, confirm that the following items are present in the figure legend, table legend, main text, or Methods section.

n/a	Confirmed	
☐	☒	The exact sample size (*n*) for each experimental group/condition, given as a discrete number and unit of measurement
☐	☒	A statement on whether measurements were taken from distinct samples or whether the same sample was measured repeatedly
☐	☒	The statistical test(s) used AND whether they are one- or two-sided<br>*Only common tests should be described solely by name; describe more complex techniques in the Methods section.*
☒	☐	A description of all covariates tested
☒	☐	A description of any assumptions or corrections, such as tests of normality and adjustment for multiple comparisons
☐	☒	A full description of the statistical parameters including central tendency (e.g. means) or other basic estimates (e.g. regression coefficient) AND variation (e.g. standard deviation) or associated estimates of uncertainty (e.g. confidence intervals)
☐	☒	For null hypothesis testing, the test statistic (e.g. *F*, *t*, *r*) with confidence intervals, effect sizes, degrees of freedom and *P* value noted<br>*Give P values as exact values whenever suitable.*
☒	☐	For Bayesian analysis, information on the choice of priors and Markov chain Monte Carlo settings
☒	☐	For hierarchical and complex designs, identification of the appropriate level for tests and full reporting of outcomes
☒	☐	Estimates of effect sizes (e.g. Cohen's *d*, Pearson's *r*), indicating how they were calculated

Our web collection on statistics for biologists contains articles on many of the points above.

Software and code

Policy information about availability of computer code

Data collection: Flow cytometry data were collected using BD FACSDiva v9. IF images using Zeiss Zen and Leica Suite and microplate data using Biotek Gen 5 version 3.09.07

Data analysis: Data were analyzed using FlowJo Version 10.9.0, Microsoft Excel version 16.77.1, GraphPad Prism Version 9.5.1, Fiji (Image J) version 2.9.0 and SnapGene version 7.1.1. Figures were compiled in Adobe Illustrator version 28.1

For manuscripts utilizing custom algorithms or software that are central to the research but not yet described in published literature, software must be made available to editors and reviewers. We strongly encourage code deposition in a community repository (e.g. GitHub). See the Nature Portfolio guidelines for submitting code & software for further information.

Data

Policy information about availability of data

All manuscripts must include a data availability statement. This statement should provide the following information, where applicable:

- Accession codes, unique identifiers, or web links for publicly available datasets
- A description of any restrictions on data availability
- For clinical datasets or third party data, please ensure that the statement adheres to our policy

All data needed to evaluate the conclusions in the paper are present in the paper and/or the Supplementary Materials. External data sources used include BioGrid, DepMap were used for data analyses. Source data are provided with this paper.

Research involving human participants, their data, or biological material

Policy information about studies with human participants or human data. See also policy information about sex, gender (identity/presentation), and sexual orientation and race, ethnicity and racism.

Reporting on sex and gender	N/A
Reporting on race, ethnicity, or other socially relevant groupings	N/A
Population characteristics	N/A
Recruitment	N/A
Ethics oversight	N/A

Note that full information on the approval of the study protocol must also be provided in the manuscript.

Field-specific reporting

Please select the one below that is the best fit for your research. If you are not sure, read the appropriate sections before making your selection.

☒ Life sciences ☐ Behavioural & social sciences ☐ Ecological, evolutionary & environmental sciences

For a reference copy of the document with all sections, see nature.com/documents/nr-reporting-summary-flat.pdf

Life sciences study design

All studies must disclose on these points even when the disclosure is negative.

Sample size	Sample sizes varied depending on the experiment. In general, for comet assays, chromosomal aberrations, microscopy images and single-molecule DNA fiber assays, at least 50 to 150 samples were counted per condition, ranging up to over 500 samples with DNA fiber assays. These were done similar to prior publication in the field for example: Hale Ncomms (https://doi.org/10.1038/s41467-023-42011-0), Lemacon NComms (https://doi.org/10.1038/s41467-017-01180-5).
Data exclusions	No data was excluded.
Replication	All experiments were performed at least three times unless otherwise stated in the figure legends.
Randomization	Randomization was not performed in this study since most experiments were based on interpreting the effects of knockdown or over-expression of particular genes, leading to varying genotypes, on a similar parent population or similarly manipulated set of cell culture cells.
Blinding	Blinding was used to independently obtain unbiased results during quantification of microscopy images and colony survival assays. Blinding was not relevant to western blots as well as machine measurement based assays as these were generated in an unbiased fashion (Western blots band intensities were analyzed by ImageJ software).

Reporting for specific materials, systems and methods

We require information from authors about some types of materials, experimental systems and methods used in many studies. Here, indicate whether each material, system or method listed is relevant to your study. If you are not sure if a list item applies to your research, read the appropriate section before selecting a response.

Materials & experimental systems

n/a	Involved in the study	
☐	☒	Antibodies
☐	☒	Eukaryotic cell lines
☒	☐	Palaeontology and archaeology
☒	☐	Animals and other organisms
☒	☐	Clinical data
☒	☐	Dual use research of concern
☒	☐	Plants

Methods

n/a	Involved in the study	
☒	☐	ChIP-seq
☐	☒	Flow cytometry
☒	☐	MRI-based neuroimaging

Antibodies

Antibodies used	The antibodies used in this work are as follows: Anti-HA 1:1000 (Sigma, H3663-200), c1orf112 1:1000 (Sigma, HPA023778), FANCD2 1:200 (Santa Cruz Biotechnology, D0114), FANCA 1:1000 (Bethyl, A301-980A), tubulin 1:1000 (Sigma, T4026-.2ML), FANCI 1:1000 (Bethyl, A301-254A), Vinculin 1:1000 (Sigma, V9131-.2ML), ATM 1:1000 (Abcam, ab81292), γH2AX (JBW301) 1:1000 (Sigma, 2884537), mouse monoclonal anti-FLAG 1:1000 (Sigma, F1804-200UG), Rabbit anti-GFP antibody 1:1000 (Abcam, ab6556), RPA32-P-S4/8 1:1000 (Bethyl, A300-245A), RPA32 1:200 (Santa Cruz Biotechnology, F0420), CHK1-P-S317 1:1000 (Cell Signaling, 2344S), CHK1 1:200 (Santa Cruz Biotechnology, I2515), FIGNL1 1:1000 (Proteintech, 17604-1-AP-150UL), GAPDH 1:8000 (Santa Cruz Biotechnology, sc-47724), ORC2 1:1000 (Abcam, ab68348), RAD51 1:1000 (Abcam, ab63801), Mouse monoclonal RAD51 1:1000 (Millipore-Sigma, Q2574784), Rad51 Antibody (H-92) 1:1000 (Santa Cruz Biotechnology, sc-8349), Goat anti-Rabbit IgG (H+L) Highly Cross-Adsorbed Secondary Antibody 1:2500, Alexa Fluor Plus 488 1:400 (Invitrogen, A32731), Goat anti-Mouse IgG (H+L) Highly Cross-Adsorbed Secondary Antibody 1:2500, Alexa Fluor Plus 488 1:400 (Invitrogen, A32723), Goat anti-Rat IgG (H+L) Highly Cross-Adsorbed Secondary Antibody, Alexa Fluor Plus 488 1:400 (Invitrogen, A48262), Goat anti-Mouse IgG (H+L) Highly Cross-Adsorbed Secondary Antibody (1:2500), Alexa Fluor Plus 594 1:400 (Invitrogen, A32742), Goat anti-Rabbit IgG (H+L) Highly Cross-Adsorbed Secondary Antibody 1:2500, Alexa Fluor Plus 594 1:400 (Invitrogen, A32740).
Validation	All experiments were done in human cell lines. Antibodies were validated by the manufacturers (see below) or using siRNA and/or CRISPR knockdown of the target proteins.<br>Anti-HA H3663-200 Sigma Supplier validation: Reactivity – Species independent activation Application – WB, IF, ICC<br>c1orf112 HPA023778 Sigma Supplier validation: Reactivity – H Application – IHC<br>FANCD2 FI17 sc-20022 Santa Cruz Biotechnology Supplier validation: Reactivity – H Application – IHC(P), WB, IP, IF<br>FANCA A301-980A Bethyl Supplier validation: Reactivity – H Application – Flow Cyt, IP, WB<br>tubulin T4026-.2ML Sigma Supplier validation: Reactivity – H R F M P R C B Application – WB, IF<br>FANCI A301-254A Bethyl Supplier validation: Reactivity – H M Application – WB, IP, IHC<br>Vinculin V9131-.2ML Sigma Supplier validation: Reactivity – H C M F C B R T Application – WB, IF, IHC (f)<br>ATM ab81292 Abcam Supplier validation: Reactivity – H Application – dot blot, Flow Cyt, WB, IHC-P, IP<br>γH2AX JBW301 05-636 Sigma Supplier validation: Reactivity – Vertebrates Application – ChIP, IHC, IC, IF, WB<br>mouse M2 anti-FLAG F1804-200UG Sigma Supplier validation: Reactivity – Species independent activation Application – IP, IF, ICH, WB<br>Rabbit anti-GFP antibody ab6556 Abcam Supplier validation: Reactivity – Species independent activation Application – IHC-P, Electron Microscopy, ICC, IP, Flow Cyt, IHC-Fr, WB<br>RPA32-P-S4/8 A300-245A Bethyl Supplier validation: Reactivity – H M Application – WB, IHC, IP, IHC-IF<br>RPA32 9H8 sc-56770 Santa Cruz Biotechnology Supplier validation: Reactivity – M R H Application – WB, IP, IF, IHC(P), ELISA<br>CHK1-P-S317 2344S Cell Signaling Supplier validation: Reactivity – H Mk Mi Application – WB<br>CHK1 G-4 sc-8408 Santa Cruz Biotechnology Supplier validation: Reactivity – M R H Application – WB, IP, IF, IHC(P), FCM, ELISA<br>FIGNL1 17604-1-AP-150UL Proteintech Supplier validation: Reactivity – H Application – WB, IF, IP<br>GAPDH sc-47724 Santa Cruz Biotechnology Supplier validation: Reactivity – H Application – WB, IF, IP, IHC(P)<br>ORC2 ab68348 Abcam Supplier validation: Reactivity – H Application – ICC/IF, WB, IHC-P, ELISA<br>RAD51 ab63801 Abcam Supplier validation: Reactivity – H Xenopus laevis Application – ICC/IF, WB<br>Mouse monoclonal RAD51 05-530-I Millipore-Sigma Supplier validation: Reactivity – H Application – IF, WB<br>Rad51 Antibody (H-92) sc-8349 Santa Cruz Biotechnology Supplier validation: Reactivity – M R H Application – WB, IP, IF, IHC(P), ELISA<br>Goat anti-Rabbit IgG (H+L) Highly Cross-Adsorbed Secondary Antibody Alexa Fluor Plus 488 A32731 Invitrogen Supplier validation: Reactivity – R Application – WB, ICC/IF<br>Goat anti-Mouse IgG (H+L) Highly Cross-Adsorbed Secondary Antibody Alexa Fluor Plus 488 A32723 Invitrogen Supplier validation: Reactivity – M Application – WB, ICC/IF<br>Goat anti-Rat IgG (H+L) Highly Cross-Adsorbed Secondary Antibody Alexa Fluor Plus 488 A48262 Invitrogen Supplier validation: Reactivity – R Application – ICC/IF<br>Goat anti-Mouse IgG (H+L) Highly Cross-Adsorbed Secondary Antibody Alexa Fluor Plus 594 A32742 Invitrogen Supplier validation: Reactivity – M Application – WB, ICC/IF<br>Goat anti-Rabbit IgG (H+L) Highly Cross-Adsorbed Secondary Antibody Alexa Fluor Plus 594 A32740 Invitrogen Supplier validation: Reactivity – R Application – ICC/IF

Eukaryotic cell lines

Policy information about cell lines and Sex and Gender in Research

Cell line source(s)	U2OS, RPE1, 293T, HeLa and SUM149 cells were obtained from ATCC.<br><br>DR-GFP U2OS cell lines were originally obtained from Dr. Maria Jasin (MSKCC, New York).
Authentication	The cells were authenticated by ATCC using STR profiling. The DR-GFP cell lines were not further authenticated.
Mycoplasma contamination	The cells were negative for Mycoplasma.
Commonly misidentified lines<br>(See ICLAC register)	No commonly misidentified cell lines were employed in this study.

Plants

Seed stocks: N/A

Novel plant genotypes: N/A

Authentication: N/A

Flow Cytometry

Plots

Confirm that:

- [ ] The axis labels state the marker and fluorochrome used (e.g. CD4-FITC).
- [ ] The axis scales are clearly visible. Include numbers along axes only for bottom left plot of group (a 'group' is an analysis of identical markers).
- [ ] All plots are contour plots with outliers or pseudocolor plots.
- [x] A numerical value for number of cells or percentage (with statistics) is provided.

Methodology

Sample preparation	Cells were fixed in ice-cold 70% ethanol (cell cycle analyses) or not (MCA, DR-GFP HR reporter assay) prior to analyses.
Instrument	BD FACSymphony A5 or BD FACS Canto II.
Software	Data was collected using BD FACSDiva™ and analyzed using FlowJo.
Cell population abundance	At least 10000 cells were analyzed in the final gated populations.
Gating strategy	Cells were gated using FSC / SSC and then for singlets using FSC-H / FSC-A prior to checking the specific flourophores.

- [ ] Tick this box to confirm that a figure exemplifying the gating strategy is provided in the Supplementary Information.
